# Relationships between Passive Sampler and Continuous Ozone (O_3_) Measurement Data in Ecological Effects Research

**DOI:** 10.1100/tsw.2001.86

**Published:** 2001-10-26

**Authors:** S. Krupa, M. Nosal

**Affiliations:** Department of Plant Pathology, University of Minnesota, St. Paul, MN 55108, USA

**Keywords:** ozone, passive sampling, continuous monitoring, probability model, concentration relationships, plant responses

## Abstract

In ecological effects research, there is a rapid increase in the application of passive sampling techniques for measuring ambient ozone (O_3_) concentrations. Passive samplers provide data on cumulative exposures of a plant to a pollutant. However, O_3_ is not an accumulative contaminant within the plant tissue, and use of prolonged passive sampling durations cannot account for the dynamics of the occurrences of O_3_ that have a significant influence on the plant response. Therefore, a stochastic Weibull probability model was previously developed and applied to a site in Washington State (1650 m MSL) to simulate the cumulative exposure data from a passive sampler, to mimic the corresponding frequency distributions of hourly O_3_ concentrations that would otherwise have been obtained by continuous monitoring. At that site the correlation between the actual passive sampler and the continuous monitor data was R2 = 0.74. The simulation of the hourly O_3_ data was based on and compared with the results obtained from a colocated continuous monitor. In this paper we report the results of the model application to data from an unrelated monitoring site (New Hampshire, 476 m MSL) with poor correlation between the passive sampling and continuous monitoring (R2 = 0.24). In addition, as opposed to the previous work, we provide comparisons of the frequency distributions of the hourly O_3_ concentrations obtained by the simulation and the actual continuous monitoring. In spite of the major difference in the R2 values, at both sites the simulation provided very satisfactory results within the 95% confidence interval, suggesting its broad applicability. The final objective of this overall approach is to develop a generic model that can simulate reasonably well the occurrences of ambient O_3_ concentrations that are dependent upon the elevation of the measurement site and the synoptic and local meteorology. Such an effort would extend the relative utility of the passive sampling data in explaining stochastic plant responses.

## INTRODUCTION

The use of passive ozone ($O_3$) samplers in ecological effects research is increasing rapidly because they are easy to use and inexpensive, and they do not require electrical power. Passive sampling systems yield cumulative exposures of a receptor to air pollutants, as their total or average concentrations integrated over the sampling period[1]. Although most recently a passive sampling duration of 12 h has been used in a program oriented toward regional-scale air-quality regulation[2], because of logistic and other considerations most plant scientists have employed 1-week[3] or 2-week[4] sampling periods. Cumulative exposures derived from prolonged sampling periods cannot account for the dynamics and flux of pollutant occurrences and the corresponding plant responses that are governed by the changing climatic patterns and physiological and growth characteristics of the plant species[5,6]. Different growth or phenomenological stages of a plant can respond differently to the same or varying $O_3$ exposure patterns. The final biomass response is a product of the stress effect minus the energy expended for repair, compensation, or avoidance[5].

Because of the aforementioned considerations, Krupa et al.[7] developed and verified a stochastic, Weibull probability model to simulate the underlying frequency distributions of hourly $O_3$ concentrations (exposure dynamics) using the single, weekly mean values obtained from a passive (sodium nitrite absorbent) sampler. The simulation was based on the data derived from a colocated continuous $O_3$ monitor. The ultimate goal of this approach is to develop a generalized model that includes elevation and main climatic factors to allow improved utility of the passive sampler data, in the absence of continuous $O_3$ monitoring, in understanding stochastic vegetation responses under ambient conditions.

## MODEL DESCRIPTION AND STEPS

There are several steps in the implementation of the model:

(1) The Empirical Cumulative frequency Distribution Function {ECDF (x)} of the hourly $O_3$ concentrations measured by a continuous $O_3$ monitor (Fig. 1) is computed.

(2) Based on the published literature[8], the cumulative distribution function of the three-parameter Weibull frequency distribution can be used as the theoretical distribution to fit the data. The Theoretical Cumulative Distribution Function, $\{TCDF(x;c,\alpha,\xi_0)\}$, is given by the formula:

$$TCDF(x;c,\alpha,\xi_0) = \int_{-\infty}^{x} c\alpha^{-1}\left[(x-\xi_0)/\alpha\right]^{c-1} \exp\left\{-\left[(x-\xi_0)/\alpha\right]^{c}\right\} dx \quad ; \qquad (\xi_0 < x) \quad (1)$$

where c is the shape of the distribution, $\alpha$ is the scale, and $\xi_0$ is the location of the highest density[9].

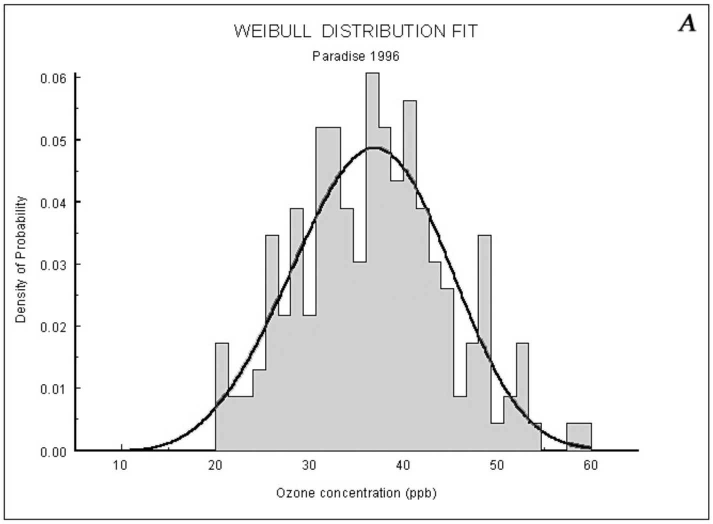

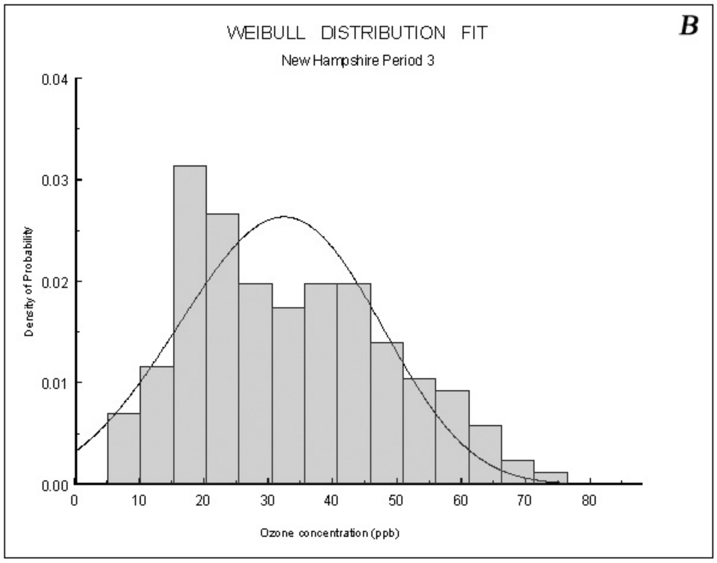

**FIGURE 1. (A)** Fit of the Weibull frequency plot for the hourly $O_3$ concentrations from continuous measurements during June 26–August 28, 1996. Although at the outset, the shape of the distribution may look normal and "bell-shaped", note the shift in the curve from left to right on the horizontal axis. **(B)** Same as (A), Mt. Washington, New Hampshire, July 19–26, 1994.

(3) In order to fit the Weibull distribution, estimation of its parameters (shape, scale, and location) is performed using the minimization of the Kolmogorov–Smirnov (KS) distribution distance. The KS distribution distance can be computed as[10]:

$$KS = \sup_{x} \left| TCDF(x; c, \alpha, \xi_0) - ECDF(x) \right| \qquad (2)$$

The best estimates of the shape, scale, and location of the Weibull distribution are values of the parameters, which minimize the KS distribution distance. The actual minimization of the KS distance is achieved using a multidimensional random search algorithm optimized by the partial derivatives of KS with respect to the estimated parameters[8].

(4) The main purpose here is to verify the theoretical, statistical methodology regardless of any practical considerations. For this reason the parameters of the best Weibull distributions obtained from continuous monitoring data in step 3 are used to generate synthetic data and their distributions compared with the distribution of the original continuous monitoring data.

(5) After obtaining best estimates of Weibull parameters for data from all passive sampling periods, a cubic regression model of those parameters can be built using the passive monitoring data as the predictor variable. This model can be written as:

$$y_i = \beta_0 + \beta_1 x + \beta_2 x^2 + \beta_3 x^3 + \varepsilon_i \tag{3}$$

where x is the passive monitoring data, y is the Weibull distribution parameter being predicted, β' values are the regression parameters, and ε' values are random error terms[11].

(6) Using the regression model from step 5, new Weibull distribution parameters are computed using the passive monitoring data as predictors. Subsequently the Weibull random-numbers generator is used to produce new synthetic data.

(7) To test the quality of the results, the cumulative distributions of the Weibull-generated synthetic data obtained in step 6 is compared with the empirical continuous $O_3$ monitoring distribution functions from step 1 using the KS distance.

## RESULTS AND DISCUSSION

Initially the model was developed using colocated passive sampling and continuous $O_3$ monitoring data collected during the growing season (June through August) of 1996 at Paradise, WA (1650 m MSL)[7]. Table 1 provides a summary of the results obtained in step 7 (comparison of the modeled data with the continuous measurements) of the model described in the previous section.

**TABLE 1**
**Summary Statistics of the Kolmogorov-Smirnov (KS) Test for Goodness of Fit Between Continuously Monitored and Synthetic Data on Hourly $O_3$ Concentrations (ppb, Weekly)**

| Period (week) | KS-distance | Sig. | Continuous $O_3$ | | Synthetic $O_3$ | | Passive $O_3$ |
|---|---|---|---|---|---|---|---|
| | | | Mean | SD | Mean | SD | Mean |
| 1 | 0.2786 | 0.000 | 36.69 | 8.00 | 32.41 | 6.41 | 24.43 |
| 2 | 0.1932 | 0.004 | 36.58 | 12.28 | 39.73 | 11.57 | 35.33 |
| 3 | 0.0917 | 0.386 | 47.68 | 12.57 | 48.14 | 11.88 | 41.27 |
| 4 | 0.2875 | 0.000 | 31.04 | 11.65 | 32.68 | 7.29 | 25.07 |
| 5 | 0.0968 | 0.391 | 54.82 | 10.47 | 53.45 | 7.90 | 49.15 |
| 6 | 0.2774 | 0.000 | 30.24 | 8.07 | 33.20 | 8.15 | 27.63 |
| 7 | 0.3626 | 0.000 | 47.54 | 11.69 | 38.47 | 10.30 | 33.14 |
| 8 | 0.1081 | 0.261 | 35.13 | 9.61 | 34.01 | 8.20 | 21.31 |
| 9 | 0.1257 | 0.126 | 48.45 | 13.50 | 51.85 | 11.42 | 44.21 |
| 10 | 0.2571 | 0.000 | 33.77 | 8.00 | 35.13 | 8.88 | 31.13 |

From: Krupa et al. [7].

*Note*: The synthetic data were generated from the frequency distribution parameters derived from the passive sampler data (Paradise, 1996).

In this particular case, compared to the passive sampler data, the mean values from the continuous monitor (Con) were always higher, ranging from 1.25 to 14.4 ppb or 3.4 to 30.3%. However, the mean values from the synthetic $O_3$ frequency distribution data (Syn) were only –3.4 to +9.07 ppb or –7.0 to +11.66% (Con·Syn) in variance from the corresponding continuous monitor data (Table 1). This is in comparison to actually observed random sampling bias of the passive sampler (–1.25 to –14.4 ppb or –3.4 to –30.3%, Table 1). The overall results predicted by the Weibull model would have been even better if the variance of the measured passive sampler mean $O_3$ concentrations had been less than what was observed, compared to the corresponding means from the colocated continuous monitor.

The model was validated by using the 1997 data from the same study site, Paradise[7]. The $R^2$ between the passive sampler and the continuous $O_3$ monitor data (Con) at Paradise was 0.72 during 1996 and 0.46 during 1997. Yet in 1997 the mean values from the synthetic $O_3$ data (Syn) derived from the mean passive sampler measurements were –3.79 to +3.03 ppb or –12.8 to +7.4% (Con·Syn) in variance from the corresponding continuous monitor data. These ranges of values were relatively similar to the original model output (–7.0 to +11.7%, Table 1) derived from the 1996 measurements. Thus, within the limitations of the passive sampler data (low $R^2$ in 1997), the model performed extremely well in simulating the hourly $O_3$ concentrations from the colocated continuous monitor[7].

To further test the performance of the model, an independent data set with a very poor correlation between the passive sampler and a colocated continuous monitor was selected ($R^2$ = 0.24), and thus data collected during July to August 1994 at Mt. Washington, NH (476 m MSL) were analyzed. Fig. 2 provides the Weibull probability plot of the hourly average $O_3$ concentrations derived from the continuous measurements. The fit of those measured values with the expected was excellent within the 95% confidence interval.

Therefore it might be concluded that the poor $R^2$ between the passive sampler and the continuous monitoring data was most likely due to the small sample size of the passive sampler data (n = 6). Also turbulent diffusion might have been a major factor at the study site, contributing to the random variance of the passive sampler performance. This hypothesis could not be tested,

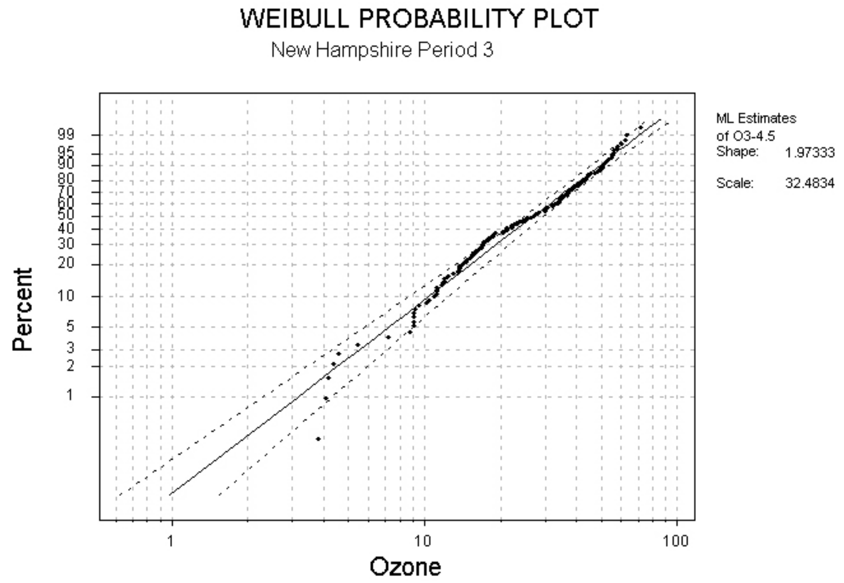

**FIGURE 2.** An example showing the Weibull probability distribution of the hourly $O_3$ concentrations (ppb) from continuous measurements during 1 week (July 19–26, 1994). The horizontal axis represents the ordered statistic of the $O_3$ levels. The solid line in the figure shows the expected, the solid circles show the distribution of the hourly concentrations, and the two dotted lines represent the 95% confidence intervals. The one data point outside the 95% confidence interval is within the 5% of occurrence of outlier values allowed in the test.

**TABLE 2**
**Summary Statistics of the Kolmogorov-Smirnov (KS) Test for Goodness of Fit Between Continuously Monitored and Synthetic Data[1] on Hourly $O_3$ Concentrations (ppb, Weekly)**

| Period[2] | KS-distance | Sig. | Continuous $O_3$ Mean | Continuous $O_3$ SD | Synthetic $O_3$ Mean | Synthetic $O_3$ SD | Passive $O_3$ Mean |
|---|---|---|---|---|---|---|---|
| 1 | 0.1964 | 0.003 | 22.00 | 10.45 | 26.75 | 10.86 | 27.37 |
| 2 | 0.1792 | 0.012 | 29.06 | 12.05 | 26.61 | 10.46 | 27.07 |
| 3 | 0.0893 | 0.515 | 33.23 | 15.39 | 31.96 | 14.35 | 29.08 |
| 4 | 0.1396 | 0.086 | 28.33 | 15.91 | 25.05 | 15.42 | 25.07 |
| 5 | 0.2024 | 0.002 | 24.97 | 18.19 | 26.82 | 14.01 | 25.53 |
| 6 | 0.1929 | 0.065 | 28.81 | 9.91 | 27.70 | 10.99 | 26.53 |

[1] Original source of raw data, Manning et al. [3], [2]Period = Week

*Note*: The synthetic data were generated from the frequency distribution parameters derived from the passive sampler data (New Hampshire, 1994).

since no wind data were available. Nevertheless, the variance between the mean continuous monitoring and the corresponding passive sampler data ranged from –3.26 to +5.37 ppb or –11.5% to +19.6% (Table 2).

Similarly the difference between the mean continuous monitoring and the corresponding passive-sampler–based synthetic data ranged from –4.75 to +3.28 ppb or –21.6 to +13.1% (Table 2). These results, in comparison to the data from Paradise (Table 1), were surprisingly reasonable given the poor correlation between the passive sampler and the continuous monitor data, further substantiating the robustness of the model.

While Table 2 contains summary statistics (weekly mean and SD) of the model output (synthetic data), Table 3 provides the results on the frequencies of the occurrences of hourly $O_3$ concentrations derived from the same synthetic data, on a weekly basis. With the exception of two extreme values during weeks 4 (late July) and 5 (early August) (Table 4), overall these distributions were relatively similar to those derived from the colocated continuous $O_3$ monitor (Fig. 3). This was in spite of the poor correlation between the original passive and the corresponding continuous monitoring data ($R^2 = 0.24$).

**TABLE 3**
**Summary Statistics of the Weibull-Generated Synthetic Hourly Ozone Frequency Distributions**

| Range ppb $O_3$ | Period[1] 1 % | 1 C%[2] | 2 % | 2 C% | 3 % | 3 C% | 4 % | 4 C% | 5 % | 5 C% | 6 % | 6 C% |
|---|---|---|---|---|---|---|---|---|---|---|---|---|
| 0-10 | 4.17 | 4.17 | 2.98 | 2.98 | 5.39 | 5.39 | 12.74 | 12.74 | 10.37 | 10.37 | 3.59 | 3.59 |
| 11-20 | 25.00 | 29.17 | 24.40 | 27.38 | 13.77 | 19.16 | 17.20 | 29.94 | 19.51 | 29.88 | 20.36 | 23.95 |
| 21-30 | 34.52 | 63.69 | 35.12 | 62.50 | 28.74 | 47.90 | 29.30 | 59.24 | 24.39 | 54.27 | 37.13 | 61.08 |
| 31-40 | 26.19 | 89.88 | 27.38 | 89.88 | 25.15 | 73.05 | 23.57 | 82.80 | 29.27 | 83.54 | 26.35 | 87.43 |
| 41-50 | 7.14 | 97.02 | 7.74 | 97.62 | 15.57 | 88.62 | 11.46 | 94.27 | 11.59 | 95.12 | 9.58 | 97.01 |
| 51-60 | 2.38 | 99.40 | 2.38 | 100.00 | 6.59 | 95.21 | 4.46 | 98.73 | 4.27 | 99.39 | 2.99 | 100.00 |
| 61-70 | 0.60 | 100.00 | | | 4.19 | 99.40 | 1.27 | 100.00 | 0.61 | 100.00 | | |
| 71-80 | | | | | 0.60 | 100.00 | | | | | | |

[1]Period = One week; [2] C% = Cumulative %

*Note:* From the New Hampshire 1994 passive sampler data.

**TABLE 4**
**Summary Statistics of the Continuous Hourly Ozone Frequency Distributions**

| Range $O_3$ ppb | Period[1] | | | | | | | | | | | |
|---|---|---|---|---|---|---|---|---|---|---|---|---|
| | 1 | | 2 | | 3 | | 4 | | 5 | | 6 | |
| | % | C%[2] | % | C% | % | C% | % | C% | % | C% | % | C% |
| 0-10 | 14.29 | 14.29 | 10.07 | 10.07 | 3.57 | 3.57 | 11.90 | 11.90 | 21.43 | 21.43 | 4.17 | 4.17 |
| 11-20 | 27.38 | 41.67 | 10.74 | 20.81 | 21.43 | 25.00 | 26.19 | 38.10 | 27.38 | 48.81 | 7.74 | 11.90 |
| 21-30 | 32.74 | 74.40 | 26.17 | 46.98 | 23.81 | 48.81 | 14.88 | 52.90 | 22.02 | 70.83 | 51.79 | 63.69 |
| 31-40 | 22.02 | 96.43 | 37.58 | 84.56 | 18.45 | 67.26 | 29.17 | 82.14 | 10.71 | 81.55 | 23.81 | 87.50 |
| 41-50 | 2.98 | 99.40 | 12.08 | 96.64 | 17.26 | 84.52 | 7.14 | 89.29 | 7.74 | 89.29 | 10.12 | 97.62 |
| 51-60 | 0.60 | 100.00 | 3.36 | 100.00 | 9.52 | 94.05 | 6.55 | 95.83 | 4.76 | 94.05 | 1.19 | 98.81 |
| 61-70 | | | | | 5.36 | 99.40 | 2.38 | 98.21 | 2.38 | 96.43 | 1.19 | 100.00 |
| 71-90 | | | | | 0.60 | 100.00 | 1.79 | 100.00 | 3.57 | 100.00 | | |

[1] Period = One week, [2] C% = Cumulative %

*Note:* From New Hampshire 1994.

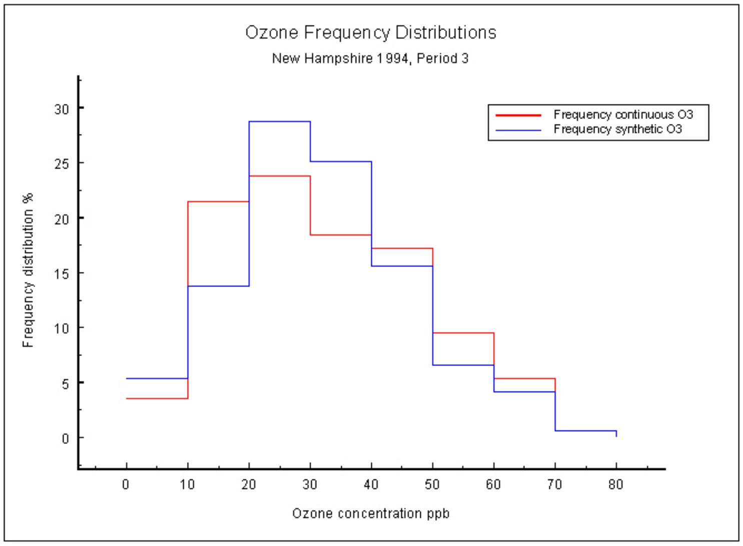

**FIGURE 3.** An example showing the relationships between the frequency distributions of hourly $O_3$ concentrations measured by continuous monitoring and synthetic data generated by the model. Mt. Washington, New Hampshire, July 19–26, 1994.

## CONCLUSION

It should be possible to improve the model output further by using multivariate statistical techniques that include climate parameters such as temperature, wind speed, relative humidity, and radiation[12,13]. That work is currently in progress. Nevertheless, it might be concluded that the overall approach might negate the initial intent of the ease and desirability of the use of passive samplers. However, as more of the needed data become available, it should be possible to develop a generic model that has wide spatial application. In that context, elevational and spatial variability in the occurrences of ambient $O_3$ concentrations must be accounted for, in relating such information to ecological effects. The present effort provides an important step beyond the normal kriging techniques (with annual or seasonal average or total $O_3$ concentrations) being used in mapping the spatial distribution of ambient $O_3$ exposures[14], and better explains the stochasticity in vegetation response relationships. With the model output, geostatistical techniques can be applied using exposure descriptors such as the weekly median and the percentiles of hourly concentrations in defining the spatial variability in the $O_3$ exposures and the corresponding differences in plant growth characteristics and responses. Furthermore, such information can be used to extend the model output to derive empirical data on the exchange of $O_3$ between the atmosphere and the plant canopy[15]. Grünhage et al.[16] have calculated flux-based (absorbed or effective dose) critical pollutant levels to protect vegetation in Europe against the adverse effects of $O_3$. Similar information is lacking in North America at the present time, although a number of investigators are examining that issue. In addition, there is a growing interest in examining diurnal vs. nocturnal $O_3$ exposure profiles in explaining the observed plant responses[17]. Exposures to pollutant mixtures can result in the maintenance of some degree of stomatal opening at night[18] and consequently the uptake of variable amounts of $O_3$ that are available at the atmosphere·plant canopy interface. In that context, there are efforts underway to develop a passive $O_3$ sampler that functions only during the daylight hours as opposed to the entire day (M. Ferm, IVL, Göteborg, Sweden, personal communication). The current model can also address those types of data if the hours of daily sunrise and sunset at the sampling location are known.

## ACKNOWLEDGEMENTS

The work described in this paper was supported in kind by the University of Minnesota Agricultural Experiment Station to the senior author. We thank Charles Barnes for his assistance in the preparation of the computer files of the tables and the figures.
